# Perioperative drug reactions – practical recommendations for allergy testing and patient management

**DOI:** 10.1007/s40629-018-0071-1

**Published:** 2018-06-04

**Authors:** Wolfgang Pfützner, Knut Brockow

**Affiliations:** 1 Department of Dermatology and Allergology, University Hospital Marburg, Baldingerstraße, 35043 Marburg, Germany; 2 Department of Dermatology and Allergology am Biederstein, Technical University of Munich, Munich, Germany

**Keywords:** Drug allergy, Perioperative, Neuromuscular blocking agents, Anesthetics, Prophylaxis

## Abstract

**Background:**

Allergy testing for perioperative drug reactions poses a particular diagnostic challenge. Neuromuscular blocking agents (NMBA) and antibiotics are among the most common triggers. In principle, however, any drug administered perioperatively is capable of causing a hypersensitivity reaction.

**Methods:**

This article is an overview of selected scientific articles and is based on research in PubMed, specialist databases, and guidelines.

**Results:**

Besides patient’s history and laboratory tests (the latter being feasible to only a limited extent), skin tests play a particularly important role. To obtain clinical relevant results, profound knowledge on the best point in time for testing, the drug concentrations to be used, how to perform tests correctly, and the assessment criteria is of special importance.

**Conclusion:**

Final outcomes of the diagnostic procedures should be providing thorough information of the patient about the findings, drugs that should be avoided in the future as well as alternative preparations, and, if necessary, preventive measures to be taken in the event of further surgical interventions.

## Introduction

Perioperative drug reactions (PODR) are defined as hypersensitivity reactions to drugs administered in immediate temporal relation to—i. e., before, during, or after—a surgical procedure. According to various studies, reactions of this kind have an incidence of approximately 1:10,000 procedures, with the number of unknown cases estimated to be much higher. They are generally immediate reactions (onset less than 1 h following administration of the triggering drug); around two thirds are immunoglobulin E (IgE)-mediated and one third is non-allergic in nature. T‑cell-mediated delayed-type reactions are rare. Due to patient history-related limitations (patient’s unawareness of reactions under general anesthesia, anesthetic log often not available or difficult to read), the multitude of different drugs administered, and the limited possibilities to test some preparations (see below), allergy testing in PODR poses a particular challenge.

## Clinical symptoms

Skin, cardiovascular, and respiratory symptoms are among those most commonly seen. Skin reactions are described less frequently in this context than in other forms of anaphylaxis. However, skin rashes such as urticaria or flushing may be concealed by surgical drapes. Cardiac symptoms such as hypotension and tachycardia can be misinterpreted as pharmacological side effects and mechanical respiratory difficulties may be attributed to insufficient sedation. Unusual reactions such as myocardial ischemia due to cardiovascular spasms or paradoxical bradycardia are also possible. Delayed reactions are seen hours to days following the surgical procedure in the form of locally indurated plaques at the injection site (e.g., delayed-type reactions to heparins), delayed urticaria and/or angioedema (e. g., as a manifestation of analgesic hypersensitivity to cyclooxygenase inhibitors), as well as delayed-type hypersensitivity rashes (particularly if drug administration is ongoing following the procedure, e. g., continued antibiotic or analgesic administration).

### Triggers

Neuromuscular blocking agents (NMBA), which can elicit both IgE-mediated and non-allergic reactions, are among the commonest triggers of PODR (approximately 60%), followed by natural rubber latex (around 20%), antibiotics (particularly beta-lactams, 15%), and volume expanders (approximately 3%). However, the relevance of latex is on the wane given the mostly abounded use of powdered latex gloves in the surgical setting. Other, to an extent far rarer, triggers include general anesthetics (e. g., propofol), opioids, non-steroidal anti-inflammatory drugs (NSAID), the disinfectant chlorhexidine, heparins, dyes, benzodiazepines, and local anesthetics. Corticosteroids can also be causative in very rare cases. The sterilant gas ethylene oxide and drug additives have also been reported eliciting PODR (Table 1).

**Table 1:** Elicitors of perioperative drug reactions

Substance group	Examples
Neuromuscular blocking agents	*Polarizing:* atracurium, cisatracurium, mivacurium, pancuronium, rocuronium, vecuronium
	*Depolarizing:* suxamethonium
Antibiotics	*Systemic:* penicillins, cephalosporins, quinolones
	*Local:* gentamicin
Analgesics (NSAID)	Paracetamol, ibuprofen, metamizole
Natural rubber latex	(Powdered) natural rubber latex gloves
Disinfectant	Chlorhexidine, povidone-iodine
Colloids	Dextrans, Gelafundin® (alpha-Gal)
Radiocontrast agents	Iotrolan, iodixanol
Opioids	Fentanyl, remifentanil, alfentanil, sufentanil, morphine
General anesthetics	Propofol, ketamine, thiopental, etomidate
Heparins	Heparin, low-molecular-weight heparins
Corticosteroids	Prednisolone
Drug excipients	Methyl cellulose, polyethylene glycol
Sedatives (benzodiazepines)	Midazolam
Local anesthetics	Lidocaine
Dyes	Patent blue, methylene blue
Sterilant gases	Ethylene oxide

*NSAID* non-steroidal anti-inflammatory drug

## Diagnostic allergy testing

### At the time of the reaction

**Fig. 1:**
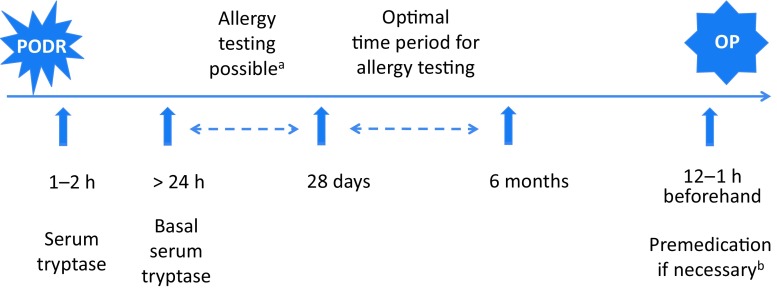
Recommended time sequence for the specialist management of perioperative drug reactions (PODR). (^a^ If it is essential for a further operative procedure (OP) to be carried out promptly, skin tests can be performed 1–28 days after the PODR, taking into consideration the possibility of false-negative results. ^b^ Prophylactic premedication is recommended particularly in cases where potential PODR triggers need to be administered again or in patients with mast cell disease, although the preventive effect is not reliable)

In the case of suspected PODR, serum to measure mast cell tryptase should be taken 1–2 h following onset of the first clinical symptoms (Fig. 1). This is elevated in up to 80% of anaphylactic reactions compared to baseline (thus the absence of elevated levels does not rule out a PODR). Increases of more than 2 ng/ml plus 20% of the basal level, which should be measured 24 h following the reaction at the earliest, are considered positive.

### Following a reaction

Skin tests, as well as in vitro and provocation tests if necessary, are used to identify the trigger. These should ideally be performed 4 weeks at the earliest, but not later than 6 months, following the PODR (Fig. 1). If earlier testing is required, e. g., in cases where a surgical procedure interrupted due to a PODR cannot be postponed for such a long period of time, positive test results are deemed conclusive. However, negative tests do not reliably exclude sensitization.

## Patient history

Every effort should be made to evaluate the anesthesia log. Approximately 90% of PODR occur during or shortly after the induction of anesthesia, but only from the anesthesia protocol is one able to determine which drugs were previously administered and in which time sequence. The surgical report can also be helpful by including, e. g., disinfectants, dyes, or materials applied locally during implantation procedures, such as gentamicin, in the diagnostic work-up. Intravenously administered triggers generally elicit clinical reactions within a few minutes, whereas topically or percutaneously administered drugs usually cause reactions with a 1- to 2‑h delay.

### Skin tests

Skin prick tests are performed in a first step to diagnose anaphylactic reactions; if negative, and to the extent that intravenous (i. v.) solutions of the drugs are available, intradermal tests are performed and readings taken after 20 min each time. Successive tests using increasing concentrations are recommended particularly for the diagnosis of more severe PODR, whereby the maximum concentrations used in skin prick tests generally correspond to undiluted drug solutions and a 1:10 dilution in intradermal tests. Exceptions are made primarily for drugs applied during induction of anesthesia, since particularly NMBA and morphine can cause false-positive test results due to their histamine-releasing properties (Table 2). A wheal diameter of at least 3 mm is regarded a positive skin prick test, while in intradermal testing, a diameter 3 mm larger than the intracutaneously administered depot of the drug solution compared with negative controls following 15–20 min is considered positive. Due to the high cross-reactivity of NMBA and the limited possibility to test these substances in provocation tests, skin testing with all preparations is recommended for this substance class in order to identify a skin test-negative NMBA as a potential alternative if the drug originally used tests positive.

**Table 2:** Maximum skin test concentrations of drugs used for anesthetic induction (from Brockow [1])

Drug (concentration)	Skin prick test (dilution factor)	Intradermal test (dilution factor)
Midazolam (5 mg/ml)	5 mg/ml (pure)	0.5 mg/ml (1:10)
Propofol (10 mg/ml)	10 mg/ml (pure)	1 mg/ml (1:10)
Ketamine (10 mg/ml)	10 mg/ml (pure)	1 mg/ml (1:10)
Thiopental (25 mg/ml)	25 mg/ml (pure)	2.5 mg/ml (1:10)
Etomidate (2 mg/ml)	2 mg/ml (pure)	0.2 mg/ml (1:10)
Morphine (10 mg/ml)	1 mg/ml (1:10)	0.01 mg/ml (1:1000)
Fentanyl (0.05 mg/ml)	0.05 mg/ml (pure)	0.005 mg/ml (1:10)
Remifentanil (0.05 mg/ml)	0.05 mg/ml (pure)	0.005 mg/ml (1:10)
Sufentanil (0.005 mg/ml)	0.005 mg/ml (pure)	0.0005 mg/ml (1:10)
Alfentanil (0.5 mg/ml)	0.5 mg/ml (pure)	0.05 mg/ml (1:10)
Atracurium (10 mg/ml)	1 mg/ml (1:10)	0.01 mg/ml (1:1000)
Cisatracurium (2 mg/ml)	2 mg/ml (pure)	0.02 mg/ml (1:100)
Rocuronium (10 mg/ml)	10 mg/ml (pure)	0.05 mg/ml (1:200)
Mivacurium (2 mg/ml)	0.2 mg/ml (1:10)	0.002 mg/ml (1:1000)
Pancuronium (2 mg/ml)	2 mg/ml (pure)	0.2 mg/ml (1:10)
Vecuronium (4 mg/ml)	4 mg/ml (pure)	0.4 mg/ml (1:10)
Suxamethonium (50 mg/ml)	10 mg/ml (1:5)	0.1 mg/ml (1:500)

To test for delayed-type reactions (e. g., local injection reaction to heparins, rash to antibiotics), a reading is generally taken of the 1:10 diluted intradermaly, as well as the undiluted epicutaneously, applied drug solutions after (1 to) 2 and 3 days (see [1]). In the case of high suspicion but negative results, further readings at 96 h and later can be helpful.

Some preparations, such as inhalation anesthetics and sterilant gases, cannot be used for skin testing.

### Laboratory tests

Although IgE diagnostic tests are commercially available for individual beta-lactams (penicillin G and V, amoxicillin, ampicillin, and cefaclor), natural latex, chlorhexidine, suxamethonium, morphine, gelatin, and alpha-1,3-galactosidase (found, for example, in Gelafundin®), their sensitivity is moderate (<60%). The detection of IgE sensitization to pholcodine as an indication of cross-reactivity due to a corresponding sensitization to NMBA or opioids is currently the subject of controversy. Cellular in vitro tests, such as the basophil activation test or the lymphocyte transformation test and the enzyme-linked immunospot (ELISpot) assay to investigate either immediate or delayed-type sensitization to possible triggers, respectively, can be helpful in some cases; however, their sensitivity and specificity are to be viewed critically [2].

### Provocation tests

Provocation tests are the gold standard of diagnostic allergy testing and every effort should be made to use them in the case of negative or equivocal laboratory and skin test results (e. g., in suspected non-allergic hypersensitivity to NSAID or local anesthetics) in order to determine drug tolerability. However, various groups of substances, such as general and inhalational anesthetics as well as NMBA, are not amenable to provocation testing due to their pharmacological activity profile. Given the potential for severe hypersensitivity reactions, these tests should generally be carried out in centers with appropriate experience in monitoring and emergency treatment.

## Practical recommendations and guidance following testing

Drugs that cause reactions in skin or laboratory tests should be avoided, unless a false-positive test reaction (e. g., to histamine liberators) can be proven by a negative provocation test. Positive skin tests or the detection of specific IgE antibodies to beta-lactams, natural rubber latex, chlorhexidine, and pyrazolone analgesics (in particular metamizole) have high predictive value. Patient history data, test findings, drugs to be avoided, and recommended alternative drugs are documented in an allergy passport, which should be presented to physicians and pharmacists in the future. Recommendations on alternative drugs are particularly important when it comes to drugs with potential cross-reactivity to structurally related preparations; their possible tolerance needs to be determined by means of negative skin tests (e. g., NMBA) or provocation tests (e. g., antibiotics and analgesics). The possibility of an increased risk for PODR to propofol in patients showing IgE-mediated sensitization to potentially cross-reacting food stuff is meanwhile discussed only in relation to children with a previous history of severe anaphylaxis to hen’s egg, but not in relation to adults or individuals allergic to soy and peanut. Similarly, there are no preventive limitations on the perioperative administration of other drugs to patients with mast cell diseases, e. g., mastocytosis, assuming they have no known history of drug hypersensitivity. The administration of morphine is an exception here.

As a precautionary measure, prophylactic premedication can be administered to these patients—as well as to patients with PODR in whom it has not been possible to unequivocally identify, and thus avoid, the trigger—prior to further surgical procedures. Although this measure is not able to reliably prevent a renewed PODR, it might prevent a course as severe as the original reaction in 90% of cases should a PODR occur. According to the recommendations on their administration in individuals allergic to radiocontrast agents, methylprednisolone 32 mg orally, for instance, can be administered 12 and 2 h prior to elective procedures or 40 mg i. v. 1–4 h prior to emergency procedures, both combined with 4 mg dimetindene i. v. 30 min beforehand. Any future surgical procedures should be performed with emergency response measures in place.

Although it is often not possible to establish the clinical relevance of positive skin tests to anesthetics, including NMBA, thereby unequivocally identifying the cause of PODR, these diagnostic measures and allergological recommendations are highly effective in the prevention of repeated anaphylactic reactions during further surgical interventions.

## Abbreviations

DA: Drug allergy
ELISpot: Enzyme-linked immunospot
IgE: Immunoglobulin E
NMBA: Neuromuscular blocking agents
NSAID: Non-steroidal anti-inflammatory drugs
PODR: Perioperative drug reaction
